# Ultraviolet Spectrophotometry of Lignin Revisited: Exploring Solvents with Low Harmfulness, Lignin Purity, Hansen Solubility Parameter, and Determination of Phenolic Hydroxyl Groups

**DOI:** 10.1021/acsomega.2c04982

**Published:** 2022-12-05

**Authors:** Jost Ruwoldt, Mihaela Tanase-Opedal, Kristin Syverud

**Affiliations:** RISE PFI AS, Høgskoleringen 6B, 7491Trondheim, Norway

## Abstract

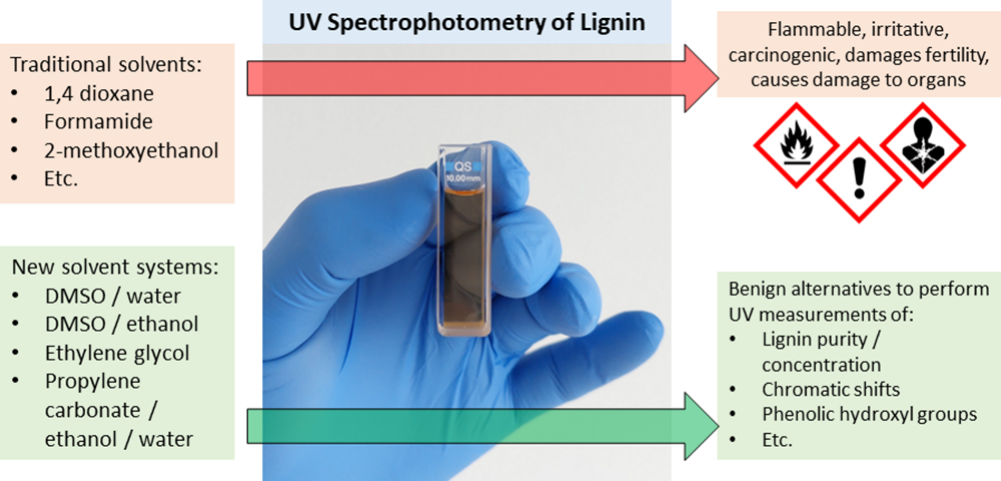

In this article, we explored solvents with lower harmfulness than established systems for UV spectrophotometry of lignin. By measuring the absorptivity in DMSO solvent at 280 nm, the purity of the lignin samples was addressed and compared with Klason and acid-soluble lignin. The general trend was an increasing absorptivity with increasing lignin purity; however, considerable scattering was observed around the sample mean. The Hansen solubility parameter (HSP) of four technical lignins was furthermore determined. The model was in line with the UV measurements, as solvents closer in HSP correlated with a higher absorptivity. Ethylene glycol was identified as a good solvent for lignin with low UV-cutoff. In addition, mixtures of propylene carbonate, water, and ethanol showed good suitability and a low cutoff of 215 nm. While DMSO itself was poorly suited for recording alkali spectra, blending DMSO with water showed great potential. Comparing three methods for determining phenolic hydroxyl units by UV spectrophotometry showed some discrepancies between different procedures and solvents. It appeared that the calibrations established with lignin model compounds may not be fully representative of the lignin macromolecule. More importantly, the ionization difference spectra were highly affected by the solvent of choice, even when using what are considered “good” solvents. At last, a statistical comparison was made to identify the most suitable solvent and method, and the solvent systems were critically discussed. We thus conclude that several solvents were identified, which are less harmful than established systems, and that the solubility of lignin in these is a crucial point to address when conducting UV spectrophotometry.

## 1 Introduction

Lignin is the second most abundant biopolymer and the greatest source of natural aromatic compounds.^1^ Due to its abundance and versatile chemistry, research has spawned a multitude of value-added applications. These include material use such as polymeric precursors and fillers,^2^ specialty chemicals such as surfactants and dispersants,^3^ fine chemicals such as vanillin,^4^ and functional micro- and nanoparticles.^5^ Knowledge of the lignin type, structure, and properties is paramount, as only this allows optimal utilization and tailoring. This article hence aims to advance lignin characterization by UV spectrophotometry.

The chemical structure of lignin is based on the three monomeric units *p*-hydroxyphenyl (H-unit), guaiacyl (G-unit), and syringyl (S-unit), which are cross-linked by various oxygen- and carbon–carbon linkages.^6^ The abundance of these monolignols depends on the type of lignocellulose biomass. Softwood lignin is essentially composed of G-units,^7^ whereas hardwood contains G- and S-units,^8^ and annual plants exhibit all three monolignols.^9^ Due to its make-up, lignin has also been described as a polyaromatic polybranched macromolecule.^3^ Naturally occurring lignin is converted to technical lignin by pulping or biorefinery operations. During pulping, the lignin is depolymerized and solubilized to be separated from the cellulose fibers.^10^ The treatment hence modifies the lignin, for example, by cleavage of the β-O-4 intermonolignol linkage. New functional groups are thereby generated such as carboxyl acid or phenolic hydroxyl groups. The abundance of phenolic hydroxyl groups is important, as this provides an indication for the reactivity and other physicochemical properties of the lignin macromolecule.^2,3^ While carboxylation may induce water solubility and thereby further the use of lignin as a dispersant,^11^ an abundance of hydroxyl groups is useful for chemical modification, as the desired functionalities may simply be added by grafting reactions.^12^ In addition, the lignin’s hydroxyl group can be utilized, e.g., as polyol replacement in polyurethanes^13^ or as cross-linkers in epoxy resins.^14^

Over the years, many characterization techniques have been developed for lignin. To determine the lignin content within a given biomass sample, it is widely established to perform acid hydrolysis and determine the acid insoluble lignin (Klason lignin) gravimetrically.^15^ The amount of acid soluble lignin can furthermore be determined by ultraviolet (UV) spectrophotometry, for example, using the absorbance at 205 nm. Implementations of this procedure are found in international standards such as TAPPI T 222, TAPPI UM 250, or ISO 24196. The functional groups of lignin can be measured by wet-chemical methods, providing measure, e.g., for the aliphatic and phenolic hydroxyl, ethylenic, carbonyl, carboxyl, and methoxy groups.^16^ Fourier-transform infrared (FTIR) spectroscopy can furthermore be employed to both characterize lignin structurally and to determine the abundance of specific functional groups.^17^ Aqueous and nonaqueous titration methods were furthermore established to measure the concentration of phenolic hydroxyl, carboxyl, and sulfonate groups.^18,19^ At last, nuclear magnetic resonance (NMR) is a powerful tool to characterize the abundance of various functionalities in lignin.^20^ Solution state NMR has been described as one of the most widely used techniques to characterize lignin.^21^

Solubility of lignin in organic solvents has been mentioned as a crucial characteristic for chemical modification.^22^ Traditional lignin solvents include, among others, dimethyl sulfoxide (DMSO), dimethyl formamide (DMF), and 2-methoxyethanol. Other lignin solvents include diethylene glycol monobutyl ether (butyl carbitol) and 1-methoxy-2-propanol (Dowanol PM).^23^ The solubility in organic solvents can also be improved, e.g., by solvent fractionation.^22,24^ Acetylation imposes acetyl groups while additionally converting hydroxyl groups to ester bonds with a lower dipole moment. Acetylated lignin has hence been shown to impart solubility in solvents such as tetrahydrofuran (THF), acetone, and even styrene.^25,26^ Lignosulfonates are also considered a form of chemically modified lignin, onto which sulfonate groups have been added. The lignosulfonate salt can readily dissociate in water at neutral pH, and solubility in water, dioxane–water mixtures, DMSO, ethylene glycol, and propylene glycol have been reported.^27^

The Hansen solubility parameter (HSP) is a useful model, which can be used for describing solvent compatibilities of polymers. In this model, three parameters are determined that describe the solubility behavior, i.e., dispersion forces (δ_*d*_), intermolecular forces (δ_*p*_), and hydrogen bonds (δ_*h*_).^28^ These parameters spawn the Hansen solubility sphere, as given in eq 1. If the distance from the sphere center (Ra) is less than the maximum distance (*Ra*_0_), i.e., , then the solubility of the polymer (index 1) in the solvent (index 2) is predicted. Albeit representing a simplified case, the HSP model is widely recognized and applied due to its straightforwardness and good predictions at low Ra values.^29,30^
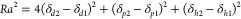
1

UV spectrophotometry is a versatile tool for characterizing lignin, as it can yield both qualitative and quantitative information.^31^ First, the absorbance has been used to quantify the concentration of lignin in solution, targeting wavelengths of, e.g., 280 or 440 nm.^32,33^ Second, procedures have been published to quantify the phenolic hydroxyl groups.^34,35^ This principle is based on shifts in the absorption spectrum of lignin, which occur due to ionization of the phenolic hydroxyl groups at alkaline conditions. By subtracting the neutral from the alkaline spectrum, the ionization difference spectrum is obtained.^31^ Different authors have correlated this difference spectrum with the abundance of phenolic hydroxyl groups in model compounds.^31,34,35^ These methods can furthermore distinguish between structures such as conjugated, saturated, and α-carbonyl. Third, chemical modification of the lignin macromolecule can be probed by UV spectrophotometry. For example, reactions that introduce unsaturated substituents on the aromatic ring reportedly yield bathochromic shifts,^31^ i.e., the absorption maximum is shifted to longer wavelengths. Blocking of phenolic hydroxyl groups, on the other hand, is said to induce hypsochromic and hypochromic changes,^36^ i.e., the absorption maximum is shifted to shorter wavelengths and lower intensity, respectively.

Solvation in a good solvent is paramount to conduct UV spectrophotometry of lignin. Previous approaches have used solvents such as formamide, 2-methoxyethanol, or 1,4-dioxane.^31,34^ These solvents have been largely replaced in today’s laboratories due to their harmful effects on humans. The goal of this study was hence to find alternatives that are less hazardous and well-suited for UV spectrophotometry of lignin. In this article, we determined the HSP of different technical lignins, exploring solvent blends that are suited for recording both neutral and alkali spectra of lignin. To provide a complete picture, the purity of lignin, solvent harmfulness, and the comparability of methods for measuring phenolic hydroxyl groups were addressed in addition.

## 2 Experimental Section

### 2.1 Materials

Solvents for UV spectrophotometry were purchased as DMSO (99,8%, anhydrous, Sigma-Aldrich), ethylene glycol (spectrophotometric grad, ≥99%, Sigma-Aldrich), ethanol (99.9%, KiiltoClean), 2-propanol (>99.9% super purity solvent, Romil Pure Chemistry), methanol (HPLC grade, ≥99.9%, Sigma-Aldrich), and propylene carbonate (ReagentPlus, 99%, Sigma-Aldrich). All solvents for testing lignin solubility were at least reagent grade at ≥98%. Distilled water was used, if not specified otherwise. For lignin analysis, 4-hydroxybenzoic acid (certified reference material, Sigma-Aldrich), acetic anhydride (ReagentPlus, ≥99%, Sigma-Aldrich), pyridine (anhydrous, >99%, TCL), and tetra-*n*-butylammonium hydroxide (TnBAH, 1 M in methanol, Sigma-Aldrich) were obtained.

An overview of all lignin samples is given in Table 1. Softwood Kraft lignin was kindly provided as BioPiva 395 (SKL1) and BioPiva 100 (SKL2) by UPM Biochemicals, Finland. In addition, softwood LignoBoost Kraft lignin (SKL3) was acquired from the Nordic Paper/RISE LignoDemo plant in Bäckhammar, Sweden. Arkansas/straw soda lignin was purchased as Protobind 1000 (ASL1), Protobind 2000 (ASL2), and Protobind 6000 (ASL3) from PLT Innovations, Switzerland. In addition, soda and organosolv lignin were produced from Norwegian spruce. The spruce soda lignin was produced according to a previously published procedure.^37^ In short, the wood chips were impregnated with an aqueous NaOH solution at a liquid/wood ratio of 7.5:1 and NaOH/wood ratio of 3:10. The liquid was circulated in a percolation autoclave, while heating to 180 °C at a rate of 1.44 °C /min and holding the final temperature for 1 h. The soda lignin was precipitated by lowering the pH with 1 M sulfuric acid to 2.5. Three batches with different purities were produced, yielding the samples SSL1, SSL2, and SSL3. The spruce organosolv lignin (SOL) was obtained by pulping with an equivolumetric mixture of acetone and water. The wood chips were immersed in cooking liquor (liquid/wood ratio 7.5:1) and heated in a batch autoclave to 195 °C at a rate of 2 K/min. The final temperature was held for 15 min. After cooling to room temperature, the cooking liquor was separated from the solids by filtration. The SOL was precipitated by adding three volumes of water per volume of cooking liquor. The precipitated lignin was filtrated, washed with water, and dried in ambient air. At last, alkali lignin (AL) was obtained from TCL, Japan.

**Table 1:** Overview of Lignin Samples Used in This Study

sample alias	botanic origin	separation process	other information
ASL1	Arkansas/straw	soda pulping	
ASL2	Arkansas/straw	soda pulping	
ASL3	Arkansas/straw	soda pulping	
SSL1	Norwegian spruce	soda pulping	high purity
SSL2	Norwegian spruce	soda pulping	ultrahigh purity
SSL3	Norwegian spruce	soda pulping	low purity
SKL1	softwood	kraft pulping	
SKL2	softwood	kraft pulping	
SKL3	softwood	kraft pulping	LignoBoost
SOL	Norwegian spruce	organosolv pulping	
AL	N/A	alkali pulping	

### 2.2 Composition and Lignin Content

The dry matter content was determined gravimetrically after drying at 105 °C for at least 3 h. The ash content was determined according to ISO 1762, i.e., the samples were combusted by heating to up to 525 °C. The acid insoluble and acid soluble lignin were determined according to TAPPI T 222 and TAPPI UM 250, respectively. No removal of extractives was done prior to acid hydrolysis.

### 2.3 Solubility

To determine the Hansen solubility parameter (HSP), 0.05 g of lignin was weighed into a vial and 10 mL of solvent was added. The vials were sealed and smoothly shaken overnight at ambient conditions. The next day, the solubility was determined by visual inspection, i.e., a soluble sample showed no solids, no turbidity, and an otherwise clear brown or dark solution. The HSP was determined from the outcome of the solubility study using the Microsoft Excel tool by Díaz de los Ríos and Hernández Ramos.^38^

### 2.4 UV Spectrophotometry

UV spectrophotometry was conducted on a Shimadzu UV-1900 UV–vis spectrophotometer. Spectra were recorded from 500 to 250 nm (or 200 nm) at 1.0 nm intervals and medium speed. The reference cell was occupied by the same blank solvent used for dilution, which was also used for recording the background spectrum. These background spectra were double checked with a new cuvette of blank solvent, where a measurement was only started if the baseline-deviation was below 0.005 cm^–1^. Stock solutions of 0.2–0.5 mg/mL lignin in blank solvent were made. Stock solutions of lignin were always freshly prepared and measured within 24 h of preparation. Furthermore, 200–600 μL of stock solution were pipetted into the quartz cuvette and diluted with 1600–2700 μL of blank solvent. The actual volumes were adjusted to yield an absorbance of 0.3–1.0 cm^–1^ at 280 nm for each measurement. Samples were run in duplicates with at least two dilutions, yielding at least four measurement points per sample. All concentrations were calculated with respect to the dry ash-free sample. The absorptivity is hence given as the absorbance divided by the dry ash-free concentration of lignin in solution.

### 2.5 Determination of Phenolic Hydroxyl Groups

#### 2.5.1 Ionization Difference Spectrophotometry

The ionization difference spectrum was calculated according to Lin & Dence,^16^ i.e., by subtracting the neutral from the alkali spectrum. Several authors have published procedures, which correlate the phenolic hydroxyl content with the ionization difference spectrum of model compounds. In the first implementation by Lin & Dence, the characteristic absorptivity maxima of the difference spectrum Δ*A*_*i*_^max^ at wavelength *i* are determined. These maxima are further used to calculate the phenolic hydroxyl *c*_phen. OH_ as stated in eq 2.^16,39^
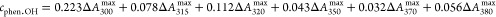
2

In the second implementation by Gärtner et al., only the absorptivity of the ionization difference spectrum at 300 (Δ*A*_300_) and 350 nm (Δ*A*_350_) is taken.^35^ It has been argued that the underlying method is more accurate in determining the phenolic hydroxyl content *c*_phen. OH_, as using 0.2 N NaOH ensured a higher pH for complete ionization.^39^
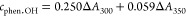
3

The third implementation was done by Chen et al. as a multipoint wavelength measurement.^34^ Here, the absorptivities at 300 (Δ*A*_300_), 320 (Δ*A*_320_), 350 (Δ*A*_350_), and 370 nm (Δ*A*_370_) are considered, calculating the contributions *c*_I_, *c*_II_, *c*_III_, and *c*_IV_ by solving the linear system of equations (eqs 3 to 7). Determination of the factors for each contribution *c_n_* was also done based on model components. The phenolic hydroxyl content *c*_phen. OH_ is lastly given as sum of the contributions, as stated in eq 8.
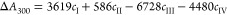
4
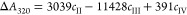
5
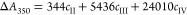
6
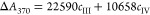
7
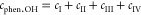
8

#### 2.5.2 Nonaqueous Titration

The procedure for nonaqueous titration of lignin was adapted from Dence and Lin,^40^ using the modified version by Gosselink et al.^18^ In this implementation, 0.15 g of lignin and 0.02 g of internal standard (4-hydroxybenzoic acid) were weighed and dissolved in 60 mL of DMSO. The sealed beaker was then titrated with 0.05 N TnBAH, which was made by diluting the stock solution (1 M TnBAH in methanol) in 2-propanol. The titrant was calibrated against 0.05 g of benzoic acid. During each run, the first inflection point at +200 to +100 mV was assigned to excess acid, whereas the second (near −350 mV) corresponded to carboxylic acids and the third (near −480 to −520 mV) to phenolic hydroxyl groups. The internal standard was subtracted from the sample each time.

#### 2.5.3 Acetylation and ATR-FTIR

Samples were acetylated by weighing 1 g of lignin into a test tube; water was removed under vacuum at 55 °C for 5 h; and 20 mL of pyridine/acetic anhydride was added at a volumetric ratio of 50:50. A total of 10 mL of DMF was added in some cases, as, for example, ASL was difficult to dissolve in the pyridine/acetic anhydride mixture. The tubes with lignin dissolved in acetylation reagent were sealed and stored at ambient conditions over silica gel for 48 h. The acetylated lignin was isolated by precipitation in distilled water, washing, filtration, and drying.

Attenuated total reflectance (ATR)–Fourier transform infrared spectroscopy (FTIR) was conducted on a PerkinElmer Spectrum 3 with a universal ATR sampling accessory. The dry lignin powder was pressed onto the ATR-crystal while the spectrum was recorded at 32 repetitions and a step rate of 4 cm^–1^. Each spectrum was baseline corrected and normalized via the aromatic stretching at 1505–1510 cm^–1^. The phenolic hydroxyl groups were determined according to Wegener & Strobel,^41^ which use the ratio of the ester peaks at 1765 and 1745 cm^–1^.

## 3 Results and Discussion

### 3.1 Lignin Purity and Relation to Absorptivity at 280 nm

The various lignin samples employed in this study are listed in Table 2. As can be seen, all samples exhibited a dry matter content between 94 and 98 wt %, which is a typical range for technical lignin. The ash content was at 1–3 wt % for most samples; however, three samples were at 0.3 wt % or below (SSL2, SKL3, and SOL), whereas AL was the highest at 17 wt %. The lignin samples from soda pulping showed a higher amount of acid-soluble lignin than the samples from Kraft or organosolv pulping. In contrast to that, the amount of acid-insoluble lignin was greater for the latter. The total lignin content was calculated as the sum of acid insoluble and acid soluble lignin. Here, values between 82.3 and 97.0 wt % were obtained.

**Table 2:** Lignin Samples and Composition

	dry matter	ash	acid-insoluble lignin	acid-soluble lignin	total lignin
sample	wt %	wt %_dry_	wt %_dry_	wt %_dry_	wt %_dry_
ASL1	94.7%	2.7%	82.4%	5.7%	88.0%
ASL2	94.7%	2.3%	77.3%	6.8%	84.1%
ASL3	95.1%	1.6%	80.2%	10.7%	91.0%
SSL1	94.9%	1.4%	88.6%	5.2%	93.8%
SSL2	96.8%	0.1%	91.4%	4.5%	95.9%
SSL3	97.2%	11.9%	74.6%	7.6%	82.3%
SKL1	95.4%	0.9%	91.7%	3.1%	94.7%
SKL2	96.3%	1.0%	91.0%	3.8%	94.8%
SKL3	95.8%	0.3%	93.5%	3.5%	97.0%
SOL	95.9%	0.2%	98.7%	1.4%	100.1%
AL	96.3%	17.0%	63.5%	18.8%	82.3%

**Figure 1:**
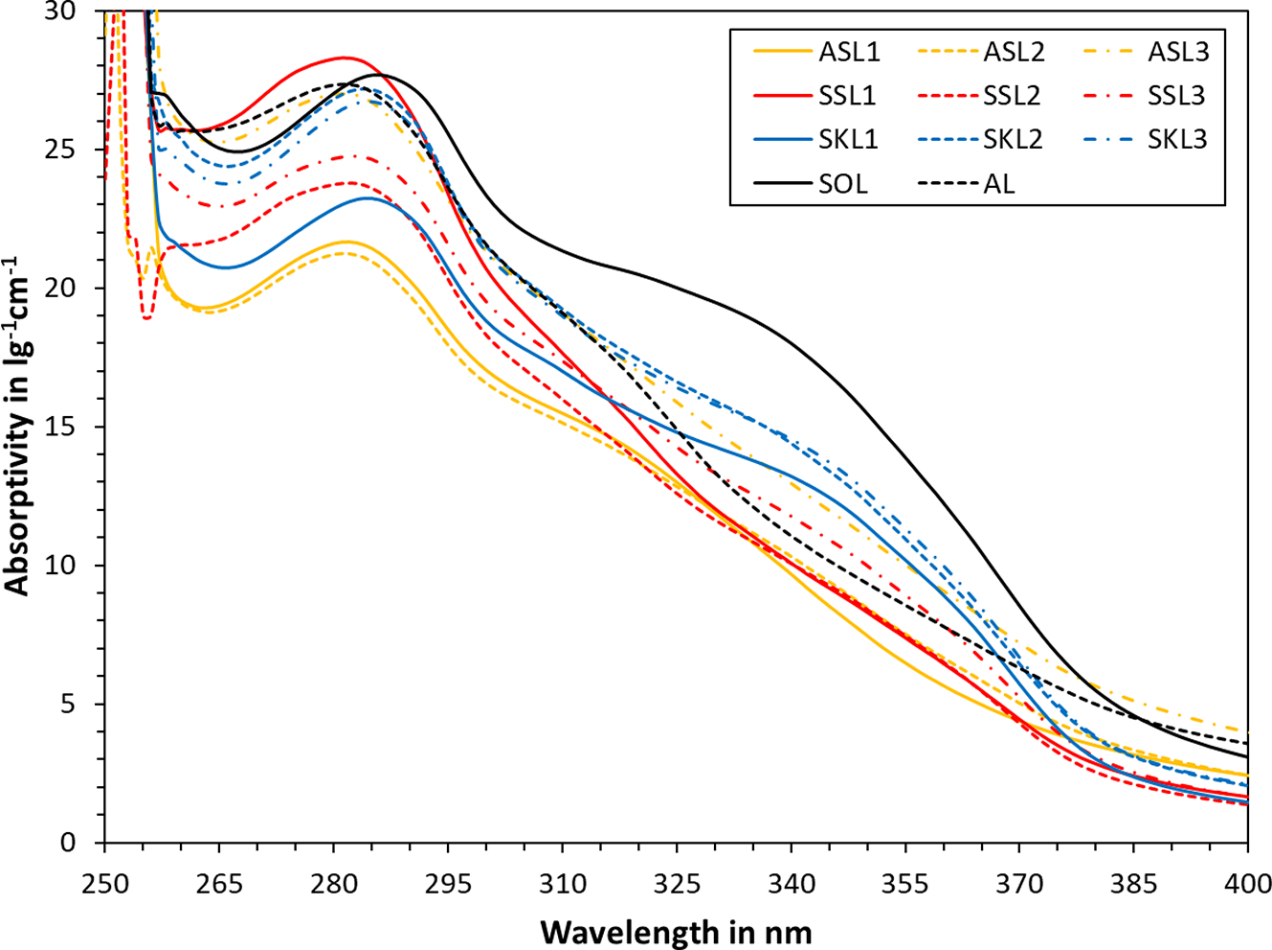
UV absorptivity spectra of various lignin samples in DMSO.

The UV spectra of various lignin samples are plotted in Figure 1. As can be seen, all samples exhibited a local maximum at around 280 nm. The actual wavelength of the maximum was shifted to the right for some of the samples. Such bathochromic shifts can be due to the monomeric structure of the lignin, for example, the presence of α-carbonyl and other ring-conjugated double bonds.^16^ This bathochromic shift is most pronounced for SKL and SOL. These samples also exhibit a shoulder at 340–355 nm. Ionization of the phenolic moieties may also induce bathochromic shifts. For all SKL and SOL samples, the natural pH of 5 wt % lignin in water dispersions was at 4.5 or below (see Supporting Information). Ionization can hence be excluded, suggesting that the observed bathochromic shift is indeed due to the chemical make-up of these samples. Below 260 nm, an increase in absorptivity and data scattering can be observed, as the UV-cutoff for the DMSO solvent was reached.

**Figure 2:**
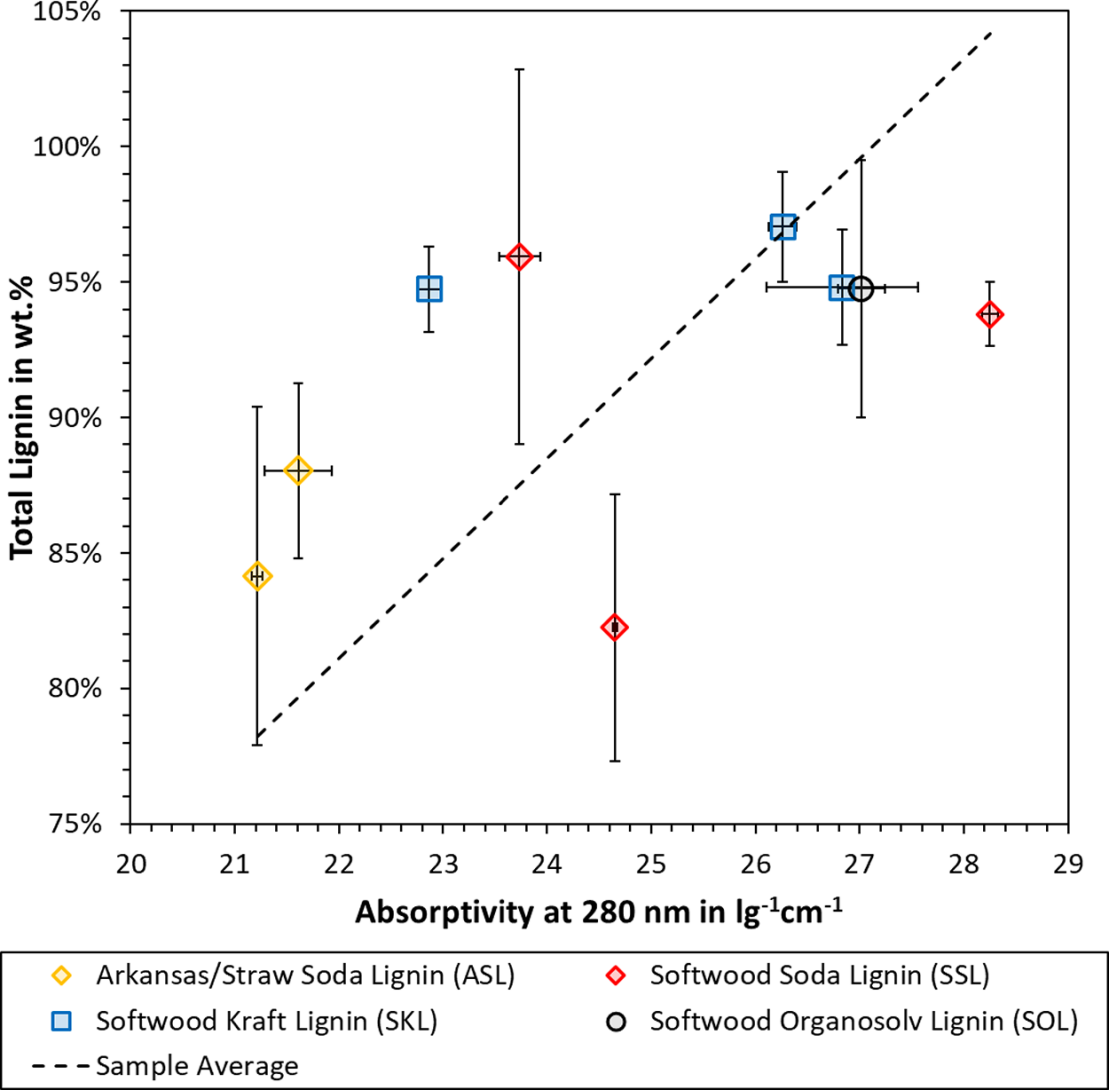
Correlation between total lignin and normalized absorptivity at 280 nm. Each point marks the average with error bars representing the standard deviation.

The absorptivity at 280 nm has been employed by many authors to quantify the lignin concentration in a specific sample or solution.^31,33,42^ The correlation between the total lignin content (acid insoluble + acid soluble) and the UV absorptivity was hence tested, as plotted in Figure 2. As can be seen, there is considerable scattering around the sample average. The standard deviation is lower for the absorptivity values than the total lignin. The latter uses gravimetric measurements and mass balancing, so it is not surprising that a spectroscopic technique exhibits better reproducibility.

Each measurement type also has specific biases. For example, sugar monomers can be condensed onto the lignin during the hydrolysis with sulfuric acid. This so-called pseudo-lignin would contribute to the acid-insoluble lignin but not necessarily to the UV absorptivity. The absorptivity, on the other hand, may be affected by impurities such as the extractives terpene and tannin.^43^ The structure and abundance of functional groups can also affect the absorptivity. In addition, the pH of the lignin solution is important, as ionization of the phenolic moieties would increase the observed absorptivity. To probe this effect, each sample was dispersed at 50 g/L in distilled water and the pH was measured. The results are listed in the Supporting Information. The pH was 4.5 or lower for all samples except ASL3 and AL, which showed a pH of 6.7 and 8.9, respectively. These two samples are hence excluded from Figure 2. According to Hubbe et al., the phenolic groups in lignin typically exhibit a pKa of 7.4–11.3.^200^ Bely et al.^200^ indeed showed that UV-spectra shifts of dioxane lignin occurred within a range of pH 5–12. It can hence be concluded that the data in Figure 2 was not affected by ionization of the phenolic hydroxyl groups.

Considering the polydispersity of the lignin macromolecule, a certain scattering around the sample average would be expected. Still, the overall trend showed a higher absorptivity with higher lignin purity. Equation 9 was derived from the sample average in Figure 2, which relates the absorptivity *A*_280 nm_ to the total lignin *p*%_lignin_. The *R*^2^ value was 0.9933, whereas the standard deviation of *p*%_lignin_ from the sample average was 7.5 wt %.
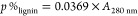
9

One assumption of eq 9 is that the observed scattering is global, i.e., independent of factors such as the sample origin and the separation process. This assumption is supported by the fact that different absorptivity values were obtained for SKL or SSL samples, which exhibited the same total lignin content. We therefore conclude that the total lignin content can be predicted from the absorptivity at 280 nm using eq 9, but this prediction involves a certain error.

### 3.2 Solubility Parameter of Technical Lignin

Four lignin samples, i.e., SKL1, SSL1, ASL1, and SOL, were tested with 27 solvents. The data can be found in the Supporting Information. In short, all tested samples were soluble in ethylene glycol, 2-methoxyethanol, pyridine, DMF, and DMSO. In addition, SOL and ASL1 were soluble in 1,4-dioxane, and SOL was soluble in PEG-400. The resulting Hansen solubility parameters (HSP) are listed in Table 3. The samples SKL1 and SSL1 were identical in terms of HSP. Both samples originate from softwood and were isolated by alkali pulping, so the observed similarity is not surprising. In comparison to this, ASL1 exhibited a lower HSP value for polar interactions. As the sample was isolated from Arkansas/straw, a higher abundance of syringyl units (S-units) can be expected than that of softwood lignin. The higher ratio of methoxy groups is likely the cause for the difference in HSP. SOL was furthermore isolated by solvent pulping, which is fundamentally different to the alkali pulping of SKL1, SSL1, and ASL1. Organosolv lignin tends to be lower in molecular mass, polydispersity, and carboxylic acid groups while exhibiting higher ratios of phenolic hydroxyl groups.^6,18,45^ Both the dispersion forces δ_D_ and the polar interactions δ_P_ of SOL were the lowest of the measured samples. The differences in chemical make-up are likely related to the lower HSP values. In addition, it makes sense that lignin from solvent pulping has an HSP closer to that of nonpolar solvents.

**Table 3:** Hansen Solubility Parameter (HSP) Determined of for Lignin Samples and Comparison with Literature Values

sample	lignin type	δ_D_	Δδ_D_	δ_P_	Δδ_P_	δ_H_	Δδ_H_
SKL1	softwood kraft lignin	17.6	2.8	12.6	7.6	15.95	20.1
SSL1	softwood soda lignin	17.6	2.8	12.6	7.6	15.95	20.1
ASL1	Arkansas/straw soda lignin	17.6	2.8	9.1	14.6	15.95	20.1
SOL	softwood organosolv lignin	16.8	4.4	9.1	14.6	15.95	20.1
ref (26)	pine kraft lignin	16.7		13.7		11.7	
ref (28)	milled wood lignin	10.85		7.0		8.8	
ref (27)	softwood lignosulfonate	∼21		13–16		∼20	

Table 3 also lists the HSP values of lignin published by other authors. As can be seen, our results are in close agreement with the HSP of pine kraft lignin.^26^ Small deviations may be evident due to differences in sample composition or tested solvents. The HSP values of milled wood lignin are lower than those of our samples,^28^ while the dispersion forces δ_D_ and hydrogen bonding δ_H_ of lignosulfonates are greater.^27^ Milled wood lignin is similar in composition to natural lignin. Lignosulfonates are chemically modified by sulfonation, which imparts water solubility and hence an HSP closer to water.

**Figure 3:**
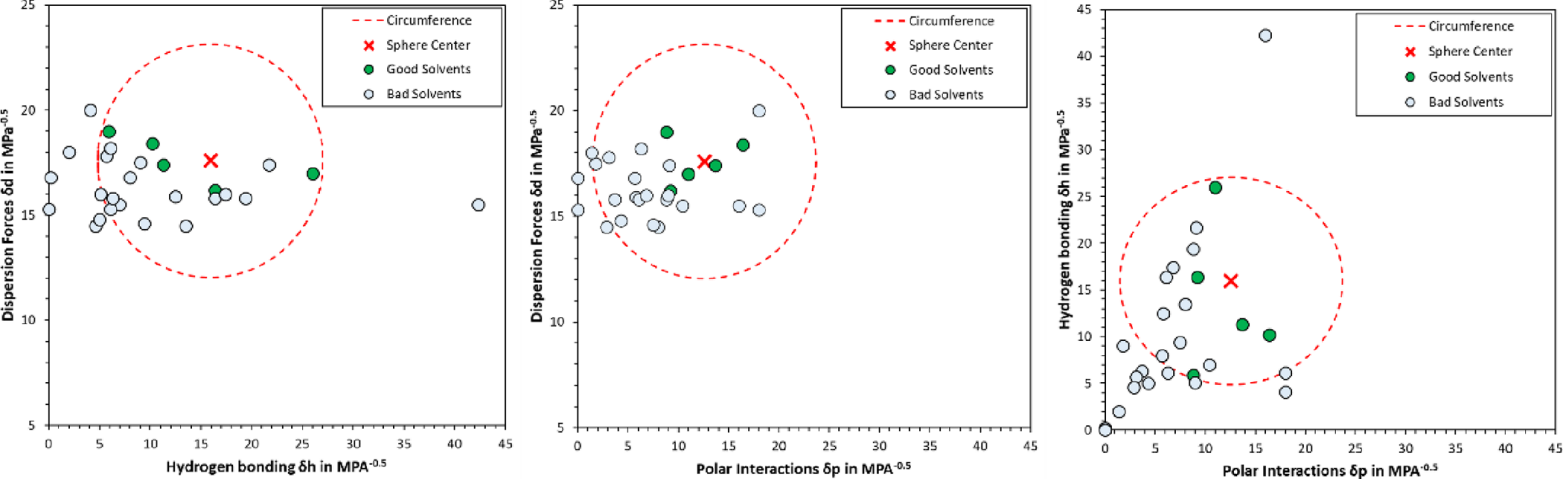
2D plots of the HSP sphere for SKL1.

The solubility sphere of SKL1 was additionally illustrated by 2D plots in Figure 3. The sphere radius *R*_0_ was determined as the distance to the outermost good solvent, i.e., including all good solvents within the sphere. This approach also led to eight “wrong in” solvents, which were included in the sphere, despite not being a good solvent. To reduce the number of “wrong in” solvents, one could reduce the sphere radius *R*_0_; however, this would also lead to the exclusion of good solvents from the sphere, i.e., increasing the number of “wrong out” solvents. Two main factors are potentially contributing to this discrepancy. First, the HSP is a simplified model, which uses only three parameters to describe solvent compatibilities. Some authors have pointed out that more complex approaches are necessary to accurately describe certain solvents.^29,46^ Second, technical lignin is a polydisperse mixture, exhibiting broad variations in the abundance of functional groups. The HSP was developed for polymers that are more homogeneous than technical lignin. In addition, components in the natural matrix may potentially interfere with the solvent efficiency.^30^

### 3.3 Effect of Solvent System on the Absorptivity of Lignin

**Figure 4:**
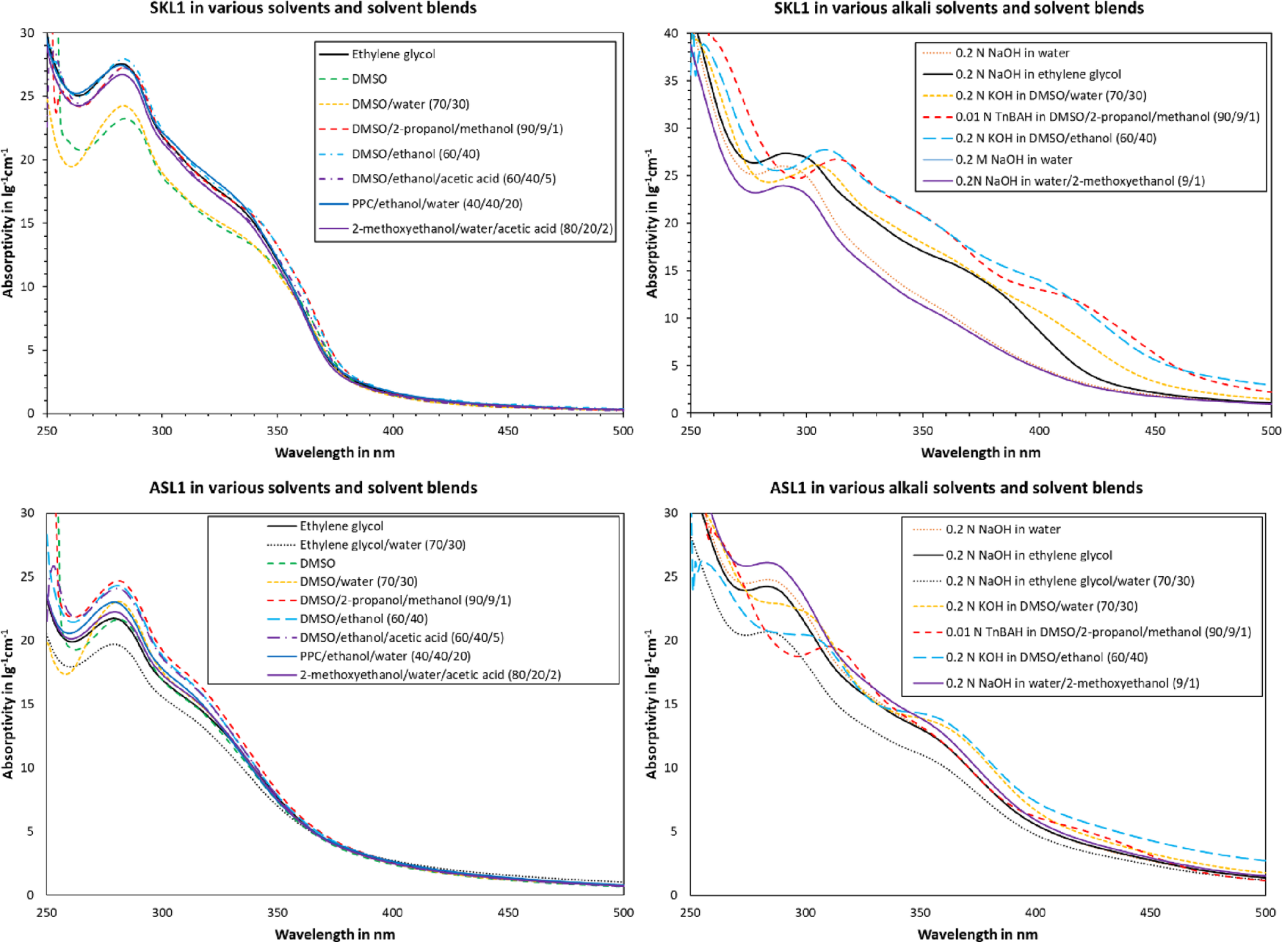
Effect of solvent on UV absorptivity of SKL1 (top) and ASL1 (bottom).

The two lignin samples SKL1 and ASL1 were selected for more detailed studies, as these originate from different pulping processes and raw materials. The effect of solvent system on the absorptivity is plotted in Figure 4. As can be seen, there are both qualitative and quantitative differences. A local maximum at 280 nm and a shoulder at 330–360 nm are visible for both SKL1 and ASL1 at low pH. In the case of SKL1 at low pH, all solvents were close to the maximum absorptivity at 280 nm, except for DMSO and DMSO/water. Mixtures of DMSO with alcohol additionally showed elevated absorptivity at the shoulder region, i.e., at 350–370 nm. For ASL1 at low pH, DMSO/alcohol mixtures accounted for the highest absorptivity. The qualitative progression only differed for ASL1 in DMSO/water, which showed a pronounced decrease at the minimum around 260 nm. The remainder of the tested solvents for ASL1 at low pH exhibited a qualitatively similar progression. Ionization by base-addition yielded more pronounced differences. A clear bathochromic shift is visible for certain solvents. For example, the local maximum at 280 nm appeared shifted to higher wavelengths. In addition, the shoulder at 350 nm exhibited a red-shift, for example in the case of SKL1 in ethylene glycol or DMSO/ethanol with added base.

To be able to describe the bathochromic shifts in quantitative terms, the local maxima at 280 nm and above were determined and listed in Table 4. As can be seen, the local maxima are located within ±1 nm of the average at 283 and 280 nm for SKL1 and ASL1, respectively. Addition of a base shifted the maximum location by 3–30 nm, depending on the solvent system. This shift appeared greater for blends of alcohol or water with DMSO (20–30 nm), whereas ethylene glycol, water, and water/2-methoxyethanol shifted the maximum by ca. 10–20 nm. It is interesting to note that the shift does not appear to correlate with the absolute absorptivity. In other words, the sample absorptivity and bathochromic shifts in alkali solvent seem independent from each other.

**Table 4:** Location of Local Maxima within the Range of 270–330 nm (Each Point Is the Average of Four Measurements with Standard Deviation)

		local maximum position
lignin	solvent	nm
SKL1	ethylene glycol	282 ± 0
SKL1	DMSO	284 ± 0
SKL1	DMSO/water (70/30)	283 ± 0
SKL1	DMSO/2-propanol/methanol (90/9/1)	284 ± 0
SKL1	DMSO/ethanol (60/40)	283 ± 0
SKL1	DMSO/ethanol/acetic acid (60/40/5)	283 ± 0
SKL1	PPC/ethanol/water (2/2/1)	282 ± 0
SKL1	2-methoxyethanol/water/acetic acid (80/20/2)	282.8 ± 0.4
SKL1	0.2 N NaOH in water	289.8 ± 0.4
SKL1	0.2 N NaOH in ethylene glycol	291 ± 0
SKL1	0.2 N NaOH in DMSO/water (70/30)	304.3 ± 0.4
SKL1	0.01 N TnBAH in DMSO/2-propanol/methanol (90/9/1)	303 ± 0
SKL1	0.2 N KOH in DMSO/ethanol (60/40)	308 ± 0
SKL1	0.2 N NaOH in water/2-methoxyethanol (9/1)	289.8 ± 0.4
ASL1	ethylene glycol	279 ± 0
ASL1	ethylene glycol/water (70/30)	279 ± 0
ASL1	DMSO	281 ± 0
ASL1	DMSO/water (70/30)	281 ± 0
ASL1	DMSO/2-propanol/methanol (90/9/1)	281 ± 0
ASL1	DMSO/ethanol (60/40)	281 ± 0
ASL1	DMSO/ethanol/acetic acid (60/40/5)	280.8 ± 0.4
ASL1	PPC/ethanol/water (2/2/1)	279 ± 0
ASL1	2-methoxyethanol/water/acetic acid (80/20/2)	279.8 ± 0.4
ASL1	0.2 N NaOH in water	283.5 ± 0.5
ASL1	0.2 N NaOH in ethylene glycol	283 ± 0
ASL1	0.2 N NaOH in ethylene glycol/water (70/30)	283 ± 0
ASL1	0.2 N NaOH in DMSO/water (70/30)	N/A
ASL1	0.01 N TnBAH in DMSO/2-propanol/methanol (90/9/1)	310.8 ± 0.4
ASL1	0.2 N KOH in DMSO/ethanol (60/40)	N/A
ASL1	0.2 N NaOH in water/2-methoxyethanol (9/1)	283.3 ± 0.5

**Figure 5:**
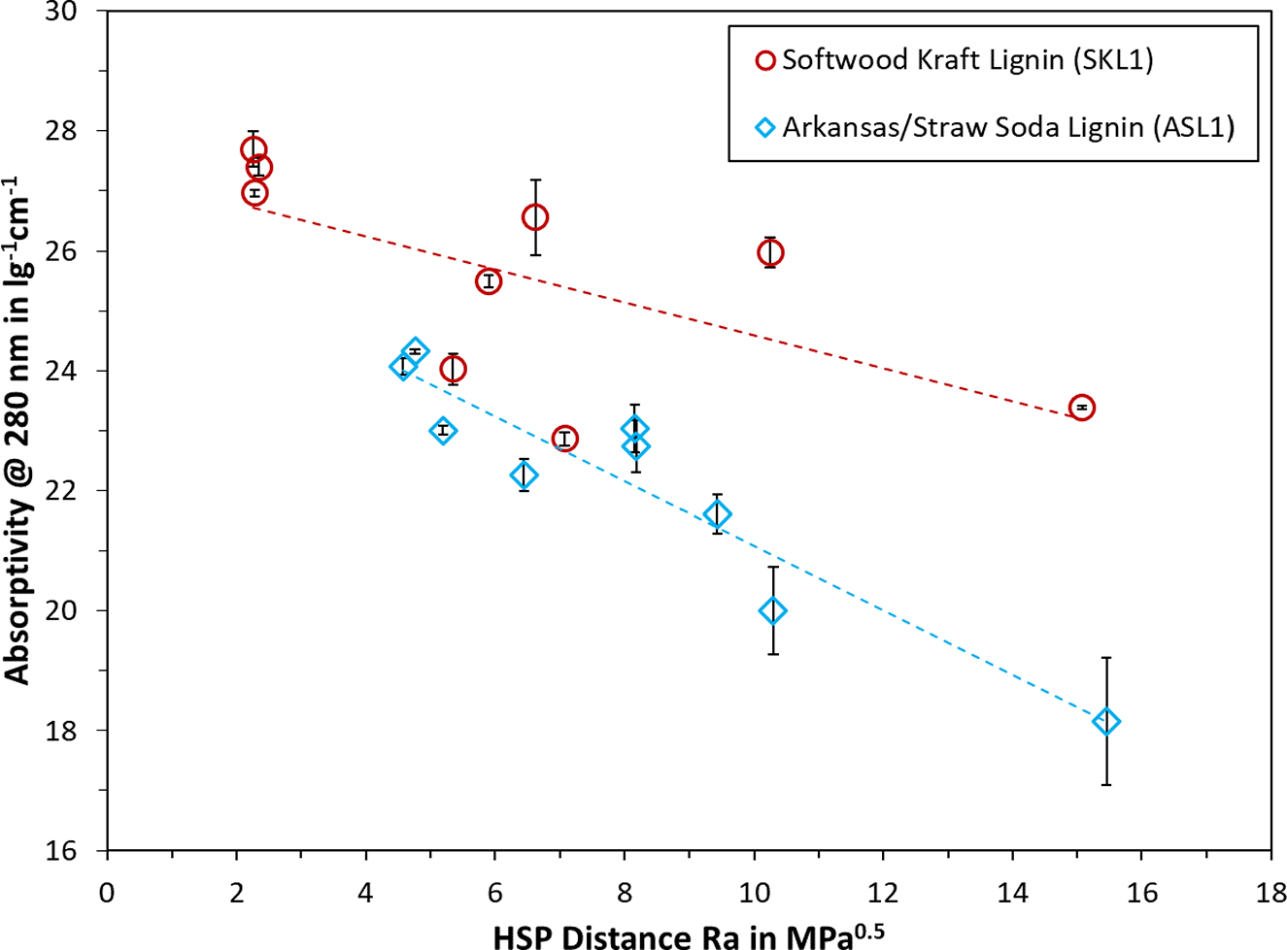
Relationship of HSP distance Ra and absorptivity of SKL1 and ASL1 in neutral solvent.

It has been indicated by other authors that a higher lignin absorptivity would be achieved with the better solvent.^16,34^ To test this concept, the absorptivity of SKL1 and ASL1 at 280 nm was plotted against the HSP distance (Ra) from the sphere center, which was calculated from the difference of solvent and lignin HSP in eq 1. The underlying theory of the HSP model is that the lower the Ra value, i.e., the lower the distance from the sphere center, the better the solvent for dissolving the polymer. The general trend can be seen in Figure 5, where a lower Ra yielded indeed a higher absorptivity. Trendlines were plotted for each data set, which show increasing tendency for lower Ra values. A certain deviation from the trendlines can be noted, which may be due to measurement error or imperfections of the HSP model. Still, as the data in Figure 5 shows, there is a good agreement between UV spectrophotometric measurements and the HSP of lignin.

### 3.4 Ionization Difference Spectra and Phenolic Hydroxyl Groups

**Figure 6:**
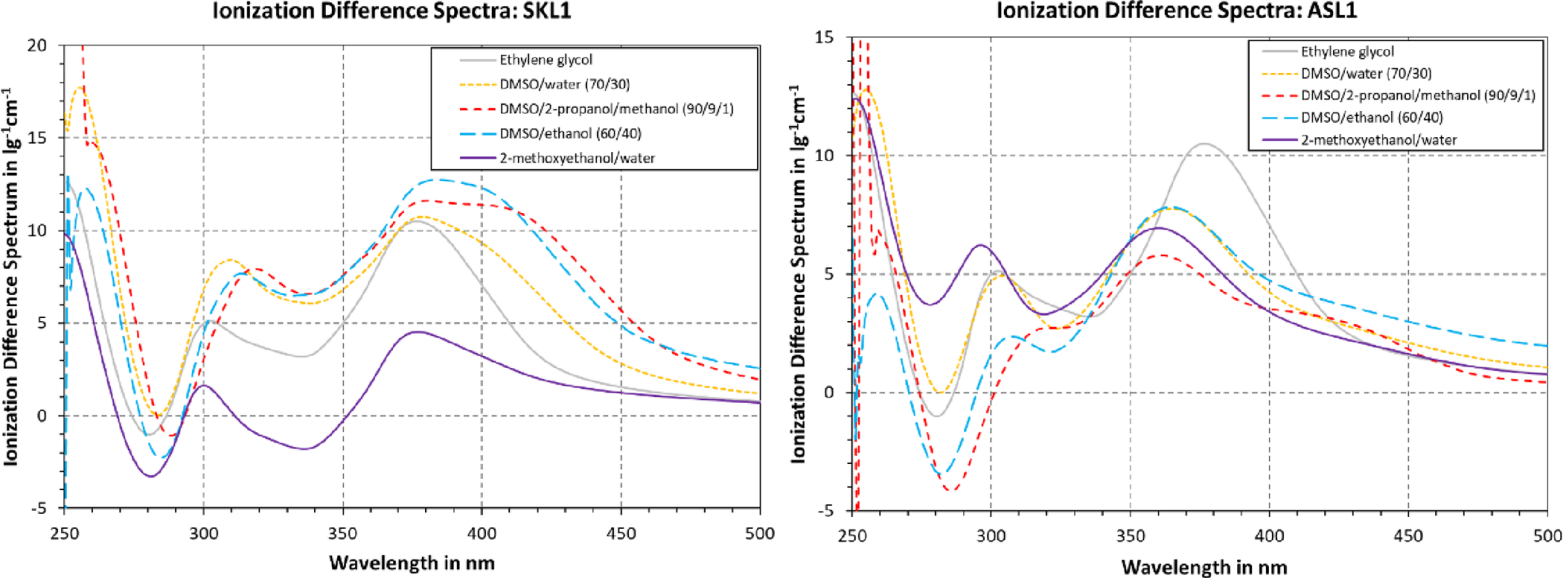
Ionization difference spectra of SKL1 and ASL1 in various solvents.

The ionization difference spectra in Figure 6 were computed based on the data from Figure 4, as this enabled identifying solvents that are suitable for both neutral and alkali UV spectrophotometry of lignin. Using the lower base concentration of 0.01 N TnBAH is in accordance with Lin & Dence,^16^ yet Goldmann et al. argued that a higher concentration of 0.2 N is necessary to ensure ionization of phenolic moieties that are difficult to ionize.^39^ When comparing the ionization difference spectrum of DMSO/2-propanol/methanol (0.01 N base) with DMSO/ethanol (0.2 N base), the difference is small compared to other samples. Still, for ASL1, the spectrum at the 0.01 N base was lower than at 0.2 N, corroborating the statement by Goldmann et al.^39^ All in all, the ionization difference spectra could be expected to coincide, as all tested solvents are considered good solvents for the technical lignin and its polyanion. Still, there are major differences in peak location and amplitude. In the case of SKL1, the qualitative progression is similar for most solvents, where one local maximum is observed between 300 and 320 nm, and a second one at 370 nm. Still, the height of the maxima differed greatly, where DMSO mixtures accounted for the highest peaks followed by ethylene glycol and 2-methoxyethanol/water mixtures at last. In the case of ASL1, the qualitative progression of the individual ionization difference spectra appeared almost arbitrary. The maximum location and height, as well as the ratio of the first to second maximum can vary. For most solvents, the first maximum appeared at around 300 nm and the second one appeared at 360 nm. Following Figure 5, the solvent systems were located further away from the HSP of ASL1 than that of SKL1. A difference in solubility behavior could potentially have contributed to the greater effect of solvent system on the ionization difference spectrum, but more data is needed to confirm this.

**Figure 7:**
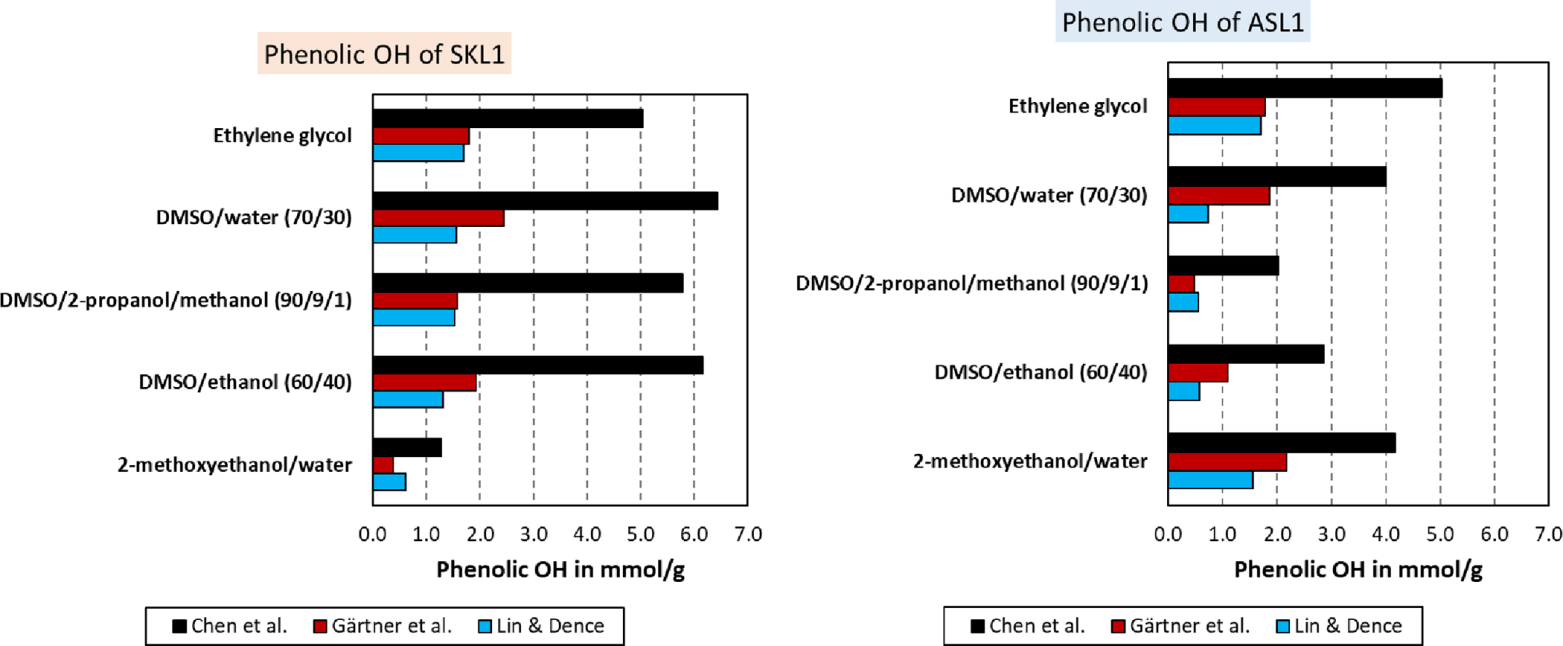
Phenolic hydroxyl content determined by UV spectrophotometry of SKL1 (left) and ASL1 (right) in various solvents and according to the procedures published by three different authors.

The ionization difference spectrum may furthermore be used to calculate the phenolic hydroxyl groups via eqs 2 to 8. The results from the three approaches are compared in Figure 7. As can be seen, the calculated phenolic hydroxyl content differed depending on the method and solvent. The methods by Lin & Dence and Gärtner et al.^16,35^ yielded concentrations between 0.4 and 2.4 mmol/g for SKL1 and 0.6 and 2.2 mmol/g for ASL1. The method by Chen et al. resulted in 1.3–6.4 mmol/g for SKL1 and 2.0–5.0 mmol/g for ASL1. This broad range of values attests a poor quality to the results, as in theory the values should agree. The phenolic hydroxyl according to Gärtner et al. is in agreement with Lin & Dence, where Gärtner et al. tended to yield on average higher values.^16,35^ The results according to Chen et al.^34^ were two to three times as much than the latter two. The overall trend, however, was the same when comparing different solvents. In other words, all three methods showed similar increases and decreases depending on the solvent system in use.

Each method has its distinctions, which may explain the results to some extent. The method by Lin & Dence uses the maxima of the ionization difference spectrum.^16^ As has been shown in Figure 6, the location and intensity of each maximum may depend on the solvent system used. Such an observation would render a principal assumption of Lin & Dence^16^ obsolete: “Based on the absorptivity maximum, the phenolic hydroxyl groups may be classified into types 1–6, i.e., structures with saturated side chains, structures with conjugated double bonds, structures with α-carbonyl groups, etc.” The fact that the same lignin sample exhibit different maximum locations, depending on the solvent system used, shows that such classification is misleading. It is hence not surprising that the phenolic hydroxyls calculated according to Lin & Dence^16^ are not converging. The method developed by Gärtner et al. does not consider the peak maxima but computes phenolic hydroxyl based on the difference spectrum at the specific wavelengths 300 and 350 nm.^35^ This approach could render the method more robust, since spectrum shifts do not alter the equations used. Still, solvent effects can potentially affect the outcome. In addition, only two data points are considered in the calculation. The spectrum of some pseudo-monomeric configurations may exhibit absorption outside the wavelengths 300 and 350 nm. Four different wavelengths were included by Chen et al.,^34^ i.e., 300, 320, 350, and 370 nm. This method would hence appear the most robust, when considering the volume of data considered.

To provide a reference for the actual phenolic hydroxyl content, a method comparison was made with nonaqueous titration and FTIR. A phenolic hydroxyl content of 3.4 mmol/g was found by nonaqueous titration for both SKL1 and ASL1. FTIR of acetylated SKL1 and ASL1 yielded 4.1 and 4.4 mmol/g, respectively. Considering this, values of within 3–5 mmol/g appear realistic. Going back to the results in Figure 7, the methods by Lin & Dence as well as Gärtner et al. did not surpass 2.4 mmol/g phenolic hydroxyl for either SKL1 or ASL1.^16,35^ Both methods therefore provided an underestimation. In the case of the method by Chen et al.,^34^ an overestimation is evident for SKL1 with all tested solvents but 2-methoxyethanol/water. It is interesting to note that this method yielded only 1.3 mmol/g with 2-methoxyethanol/water, since the method was originally calibrated with exactly this solvent and guaiacyl-type lignin, such as SKL1. In the case of the Arkansas/straw lignin ASL1, the method by Chen et al.^34^ was closest to the values measured by nonaqueous titration and FTIR. The solvent systems DMSO/water and 2-methoxyethanol/water yielded values of 4.0 and 4.2 mmol/g, respectively, which agrees with FTIR. Still, other solvents would vary from 2.0 to 5.0 mmol/g, hence indicating a considerable experimental error. To provide a more statistical approach for correlating the data, eq 10 was applied. Here, the sum of squared differences (SSD) was computed, where *c*_*i*, UV_ is the content of phenolic hydroxyl groups determined by UV spectrophotometry of lignin *i*, whereas *c*_*i*, *j*_ is the content of phenolic hydroxyl groups as determined by method *j*, i.e., titration or FTIR.
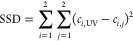
10

The SSD values for various methods and solvents are compared in Table 5. The advantage of this approach is that it provides an overview over the total deviations. In addition, the sum for each method or solvent is listed at the end of the rows or columns, respectively. Of all three methods, the approach by Chen et al.^34^ yielded the best agreement with the two other techniques, exhibiting the smallest SSD values on average. Ethylene glycol in combination with Chen et al.^34^ showed the lowest overall SSD. Ethylene glycol furthermore exhibited the lowest SSD for Lin & Dence,^16^ and the second lowest SSD for Gärtner et al.^35^ The second-best solvent according to Table 5 would be DMSO/water at 70/30. Both solvent systems include a good lignin solvent (DMSO or ethylene glycol) and a solvent with a high dipole moment (water or ethylene glycol), which would be necessary to dissolve ionized compounds.

**Table 5:** Sum of Squared Differences (SDD) for Various Methods and Solvents

solvent/reference	Chen et al.^34^	Gärtner et al.^35^	Lin & Dence^16^	sum
ethylene glycol	6.6	17.4	18.8	42.8
DMSO/water (70/30)	15.0	12.5	30.3	57.8
2-methoxyethanol/water	14.4	23.1	34.8	72.3
DMSO/2-propanol/methanol (90/9/1)	13.2	29.5	31.6	74.3
DMSO/ethanol (60/40)	16.0	33.7	32.8	82.5
sum	51.9	86.6	116.9	

Overall, the ionization difference spectra were largely divergent, despite using good solvents for the same lignin sample. It appears that solvent effects influence both the location and intensity of peaks in these spectra. As a result, all three approaches for determining phenolic hydroxyl by UV spectrophotometry disagreed, both to each other and for the same method with different solvents. The methods by Lin & Dence or Gärtner et al. yielded values that were consistently 1–2 mmol/g lower than the phenolic hydroxyl content determined by titration or FTIR.^16,35^ All UV methods were based on the ionization difference spectrum of model compounds. One explanation for the observed discrepancies is that the chosen model compounds are not fully representative of the polydisperse lignin macromolecule. Moreover, none of the methods addressed solvent compatibilities, which may vary depending on lignin origin and separation process. The choice of solvent can hence affect the observed absorptivity and thereby the ionization difference spectrum. At the bottom line, several solvent systems for UV spectrophotometry of lignin were identified and evaluated. According to our results, the model by Chen et al.^34^ with ethylene glycol as solvent was in closest agreement with the other two techniques for determining phenolic hydroxyl groups.

### 3.5 Discussion of Solvent Selection

To be able to objectively assess the harmfulness of the tested solvents, a harmfulness rating was devised, as depicted in Table 6. Here, one point was assigned per fulfilled category, which included flammability, corrosiveness, carcinogenicity/damage to organs, damage to the reproductive system, hazard upon touching, hazard upon inhalation of vapors, and splashing hazard (eye irritation, etc.). Fulfillment of these criteria was evaluated based on the HSE datasheets of each individual component in accordance with EC no. 1907/2006 – REACH. A theoretical score of 0–7 was possible, where lower values accounted for a lower potential harmfulness. The number-based rating was translated to wording via the following key: low (0–2), medium (3), elevated (4), high (5), and very high (6–7). As can be seen, DMSO and ethylene glycol, as well as their blends with water, were attributed with the lowest harmfulness. In the case of alkali solutions, water or DMSO/water blends were the least concerning. It should be mentioned that none of the tested solvents were entirely free of hazards; however, the goal of this rating was to support the identification of less dangerous systems. The advantages and shortcomings of individual solvents and solvent blends will be discussed more in detail below.

**Table 6:** Overview of UV-Cutoff of Various Solvent Systems and their Harmfulness Rating^a^

solvent system	UV cutoff^a^	harmfulness rating
DMSO	260	low
DMSO/water (70/30)	250	low
DMSO/2-propanol/methanol (90/9/1)	260	elevated
DMSO/ethanol (60/40)	255	medium
DMSO/ethanol/acetic acid (60/40/5)	255	high
ethylene glycol	210	low
ethylene glycol/water (70/30)	205	low
PPC/ethanol/water (2/2/1)	215	medium
2-methoxyethanol/water/acetic acid (8/2/0.2)	230	very high
0.2 N NaOH in water	220	medium
0.2 N KOH in DMSO/water (70/30)	250	medium
0.01 N TnBAH in DMSO/2-propanol/methanol (90/9/1)	260	high
0.2 N KOH in DMSO/ethanol (60/40)	255	high
0.2 N NaOH in ethylene glycol	220	elevated
0.2 N NaOH in ethylene glycol/water (70/30)	220	elevated
0.2 N NaOH in water/2-methoxyethanol (90/10)	225	very high

a Highest cutoff measured for SKL1 and SL1; values rounded up to the next multiple of 5.

Dimethyl sulfoxide (DMSO) is a commonly established lignin solvent. While DMSO itself exhibits low toxicity, combinations with other toxic agents present a risk, as DMSO easily penetrates the skin and other membranes.^48^ UV spectrophotometry-related limitations include a UV cutoff at 260 nm and poor solubility of NaOH, which is commonly used in ionization difference spectrophotometry. In part due to these limitations, several alternatives were explored as listed in Table 6. In general, a UV cutoff as low as 200 nm is desirable, as this enables full resolution of the characteristic peaks of lignin.

One solution to the low solubility of NaOH in DMSO was the use of a different base. Tetra-*n*-butylammonium hydroxide (TnBAH) is traditionally used during nonaqueous titration of lignin in solvents such as dimethylformamide (DMF)^18^ and has good solubility in DMSO. A downside of TnBAH is its harmfulness, as “flammable liquid” is listed by ECHA in addition to “causes severe skin burns and eye damage”.^47^ Moreover, the TnBAH stock solution used in this study was delivered in methanol, which can pose additional safety hazards. Potassium hydroxide (KOH) was identified as an alternative in this study, as it possesses better solubility in DMSO than NaOH and is less dangerous than TnBAH. The use of co-solvents enabled solutions of 0.2 N KOH in DMSO, i.e., addition of 30 vol % water or 40 vol % ethanol as described in our experiments. A ratio of 70/30 DMSO/water was used, as 0.2 N KOH were not soluble in 90/10 DMSO/water, whereas the lignin solubility was limited at 50/50 DMSO/water. Adding water furthermore decreased the UV-cutoff to 250 nm. The ratio was further extended to 60/40 for DMSO/ethanol, because ethanol has a lower dipole moment than water. As HSP calculations showed, this blend was a better solvent for lignin than DMSO alone. By minimizing the Ra value, the theoretical optimum would be at 47/53 DMSO/ethanol for SKL1 or 31/69 for ASL1. Based on our experience, blends of DMSO and water or ethanol are hence convenient and benign alternatives, which offset some of the disadvantages of DMSO alone.

Water as a solvent is well-suited for UV measurements due to a cutoff below 200 nm. However, only modified lignin, e.g., by sulfonation or carboxylation, is water-soluble at neutral pH. Two alternatives for UV spectrophotometry at wavelengths below 260 nm were identified. First, ethylene glycol is a good lignin solvent with a cutoff of 210 nm. The harmfulness of ethylene glycol is also very low, if not ingested. The only downside is a high viscosity, which can make diluting and accurate volumetric dosing difficult. Measurements with ethylene glycol exhibited the largest experimental error, which is likely related to the high viscosity. Blends of ethylene glycol/water (70/30) were hence also tested, where the water was added as a viscosity reducer. These blends showed a better reproducibility than ethylene glycol alone; however, the solubility of lignin was limited. The second alternative was propylene carbonate (PPC) mixed with ethanol and water. As our experiments showed, ethanol can also be substituted by 2-propanol. The HSP of a three-component mixture is given as the sum of individual contributions times their volume fraction.^49^ An optimum can hence be calculated as linear combination of the HSP of each solvent. This linear system of equations was neither overdetermined nor underdetermined and hence yielded a single solution, as all vectors were linearly independent. For SKL1, this solution predicted an optimum of 34/62/6 PPC/ethanol/water or 40/44/17 PPC/2-propanol/water. A ratio of 2/2/1 PPC/ethanol/water was chosen for the sake of simplicity. This blend exhibited indeed good lignin solubility, a UV-cutoff of 215 nm, a sufficiently low viscosity, and low harmfulness. The only limitation is the use of strong bases, as adding NaOH or KOH led to the formation of white precipitate, likely as a result of chemical reactions involving PPC.

The addition of bases enables the use of water as solvent, as phenolic moieties are ionized. For 0.2 N NaOH in water, ethylene glycol, or blends thereof, the observed UV-cutoff was the lowest at 220 nm. At 250 nm and above, no difference was observed when comparing the same solvent with or without base. The lowest harmfulness rating was attributed to water, ethylene glycol, DMSO, and blends thereof, as there are virtually no hazards listed by ECHA.^49^ Adding ethanol slightly increased the rating, as it is considered a flammable liquid and vapor. Acetic acid is a flammable liquid and vapor and can in addition cause severe skin burns and eye damage, hence elevating the harmfulness rating to high. Methanol also increased the rating, as it can be toxic if inhaled. Adding bases also generally increased the hazard due to their corrosive nature. The highest rating was assigned to mixtures including 2-methoxyethanol, as this may damage fertility, is harmful if inhaled, and causes damage to organs.

In conclusion, several alternatives to traditional solvents for UV spectrophotometry were found. While DMSO is a good-working lignin-solvent with low toxicity, its UV-cutoff at 260 nm is limiting. Mixtures of PPC, ethanol, and water showed potential due to a lower cutoff at 215 nm, and since these mixtures are comparably benign. Another alternative is given by ethylene glycol or blends thereof with water, which predicted the phenolic hydroxyl content of lignin in the closest agreement with other techniques. For ionization difference spectrophotometry, blends of DMSO with water were the most promising in terms of handling and low harmfulness.

## 4 Conclusions

This article summarizes our efforts to identify solvents with lower harmfulness for UV spectrophotometry of lignin, which may furthermore be used to measure phenolic hydroxyl by ionization difference spectrophotometry.

The absorptivity at 280 nm was on average greater for lignin samples with higher purity, but the experimental error remained substantial. The difference in HSP of lignin and solvent (HSP distance Ra) furthermore correlated with this absorptivity, i.e., better solvents yielded a higher absorptivity. The HSP model was hence in line with the UV measurements. Blends of DMSO and ethanol were equivalent to established solvents, such as 2-methoxyethanol, and superior to 2-methoxyethanol/water mixtures. The choice of solvent affected both neutral and alkali spectra, where the latter could vary in both absorbance and peak location. Because of this, ionization difference spectra were greatly affected by the solvent of choice, even for “good” solvents. The model by Chen et al.^34^ in combination with ethylene glycol measured the phenolic hydroxyl content, which was in the closest agreement with the other two techniques, i.e., nonaqueous titration and FTIR.

In conclusion, the observed amount of phenolic hydroxyl groups can depend not only on the lignin sample but also on the solvents involved. Solvent compatibility is hence an important factor, which should be addressed when conducting UV spectrophotometry of lignin. DMSO, ethylene glycol, or mixtures of propylene carbonate, ethanol, and water were identified as less hazardous alternatives to traditional lignin solvents in UV spectrophotometry. For measuring ionization difference spectra, ethylene glycol or blends of DMSO and water appeared the moist suited.

## Supporting Information Available

Natural pH of 5 wt % aqueous lignin dispersions (Table S1); binary solubility data of the tested lignin samples in various solvents (Table S2); and Hansen solubility parameters of various solvents employed in this study (Table S3) (PDF)
