# Supplementary material for: Ultraviolet Spectrophotometry of Lignin Revisited: Exploring Solvents with Low Harmfulness, Lignin Purity, Hansen Solubility Parameter, and Determination of Phenolic Hydroxyl Groups
Source: ACS Omega. 2022 Dec 5;7(50):46371–83. doi: 10.1021/acsomega.2c04982 (PMC9773929; doi:10.1021/acsomega.2c04982)
Supplement: Supplementary file 1 — ao2c04982_si_001.pdf [file ao2c04982_si_001.pdf]

Supporting Information for

UV spectrophotometry of lignin revisited: Exploring solvents with low harmfulness, lignin purity, Hansen Solubility Parameter, and determination of phenolic hydroxyl groups

Jost Ruwoldt[a*], Mihaela Tanase-Opedal[a], Kristin Syverud[a]

[a]RISE PFI AS, Høgskoleringen 6B, 7491 Trondheim, Norway

*Corresponding author email: jostru.chemeng@gmail.com

Table S1. Natural pH of 5 wt.% lignin dispersion in distilled water

Alias	Lignin type	pH
ASL1	Arkansas/straw Soda Lignin	3.6
ASL2	Arkansas/straw Soda Lignin	3.7
ASL3	Arkansas/straw Soda Lignin	6.7
SKL1	Softwood Kraft Lignin	2.4
SKL2	Softwood Kraft Lignin	4.5
SKL3	Softwood Kraft Lignin	3.2
SSL1	Spruce Soda Lignin	4.3
SSL2	Spruce Soda Lignin	3.1
SSL3	Spruce Soda Lignin	3.7
SOL	Spruce Organosolv Lignin	3.4
AL	Alkali Lignin	8.9

Table S2. Binary solubility data of the tested lignin samples

Solvent	SKL1	SSL1	ASL1	SOL
Water	0	0	0	0
Ethanol	0	0	0	0
1-Propanol	0	0	0	0
2-Propanol	0	0	0	0
1-Hexanol	0	0	0	0
Ethylene glycol	1	1	1	1
1,4 Dioxane	0	0	1	1
PEG-400	0	0	0	1
2-Methoxyethanol (Cellosolve)	1	1	1	1
Acetone	0	0	0	0
2-Butanone (MEK)	0	0	0	0
Acetic acid (glacial)	0	0	0	0
Diethyl ether	0	0	0	0
Metyl tert-butyl ether	0	0	0	0
n-Butyl acetate	0	0	0	0
Propylene carbonate	0	0	0	0
Propylene glycol	0	0	0	0
Dichloromethane (DCM)	0	0	0	0
Chloroform	0	0	0	0
Tetrahydrofuran (THF)	0	0	0	1
Pyridine	1	1	1	1
Toluene	0	0	0	0
Cyclohexane	0	0	0	0
n-Heptane	0	0	0	0
Acetonitrile	0	0	0	0
Dimethyl formamide (DMF)	1	1	1	1
Dimethyl Sulfoxide (DMSO)	1	1	1	1

Table S3. Hansen Solubility Parameters of various solvents employed in this study.

Solvent	Dispersion δd *MPa*$^{0.5}$	Polar interactions δp *MPa*$^{0.5}$	Hydrogen bonding δh *MPa*$^{0.5}$
Water	15.5	16.0	42.3
Ethanol	15.8	8.8	19.4
1-Propanol	16	6.8	17.4
2-Propanol	15.8	6.1	16.4
1-Hexanol	15.9	5.8	12.5
Ethylene Glycol	17	11	26
1,4-Dioxane	17.5	1.8	9
PEG -400	14.6	7.5	9.4
2-Methoxyethanol (Cellosolve)	16.2	9.2	16.4
Acetone	15.5	10.4	7
2-Butanone (MEK)	16	9	5.1
Acetic acid (glacial)	14.5	8.0	13.5
Diethyl Ether	14.5	2.9	4.6
Metyl tert-Butylether	14.8	4.3	5
Propylene Carbonate	20	18	4.1
Dichloromethane (DCM)	18.2	6.3	6.1
Chloroform	17.8	3.1	5.7
Tetrahydrofuran (THF)	16.8	5.7	8
Pyridin	19.0	8.8	5.9
Toluene	18	1.4	2
Cyclohexane	16.8	0	0.2
n-Heptane	15.3	0	0
Acetonitrile	15.3	18	6.1
N,N-Dimethyl Formamide (DMF)	17.4	13.7	11.3
Dimethyl Sulfoxide (DMSO)	18.4	16.4	10.2
